# Examining the Effects of Socioeconomic Development on Fine Particulate Matter (PM2.5) in China’s Cities Based on Spatial Autocorrelation Analysis and MGWR Model

**DOI:** 10.3390/ijerph20042814

**Published:** 2023-02-05

**Authors:** Yanzhao Wang, Jianfei Cao

**Affiliations:** 1 College of Geography and Environment, Shandong Normal University, Jinan 250014, China; 2 Shandong Dongying Institute of Geographic Sciences, Dongying 257000, China

**Keywords:** PM2.5 concentration, spatial autocorrelation, multiscale geo-weighted regression, spatial heterogeneity, scale effect

## Abstract

Understanding the characteristics of PM2.5 and its socioeconomic factors is crucial for managing air pollution. Research on the socioeconomic influences of PM2.5 has yielded several results. However, the spatial heterogeneity of the effect of various socioeconomic factors on PM2.5 at different scales has yet to be studied. This paper collated PM2.5 data for 359 cities in China from 2005 to 2020, as well as socioeconomic data: GDP per capita (GDPP), secondary industry proportion (SIP), number of industrial enterprise units above the scale (NOIE), general public budget revenue as a proportion of GDP (PBR), and population density (PD). The spatial autocorrelation and multiscale geographically weighted regression (MGWR) model was used to analyze the spatiotemporal heterogeneity of PM2.5 and explore the impact of different scales of economic factors. Results show that the overall economic level was developing well, with a spatial distribution trend of high in the east and low in the west. With a large positive spatial correlation and a highly concentrated clustering pattern, the PM2.5 concentration declined in 2020. Secondly, the OLS model’s statistical results were skewed and unable to shed light on the association between economic factors and PM2.5. Predictions from the GWR and MGWR models may be more precise than those from the OLS model. The scales of the effect were produced by the MGWR model’s variable bandwidth and regression coefficient. In particular, the MGWR model’s regression coefficient and variable bandwidth allowed it to account for the scale influence of economic factors; it had the highest adjusted R^2^ values, smallest AICc values, and residual sums of squares. Lastly, the PBR had a clear negative impact on PM2.5, whereas the negative impact of GDPP was weak and positively correlated in some western regions, such as Gansu and Qinghai provinces. The SIP, NOIE, and PD were positively correlated with PM2.5 in most regions. Our findings can serve as a theoretical foundation for researching the associations between PM2.5 and socioeconomic variables, and for encouraging the coequal growth of the economy and the environment.

## 1. Introduction

For many years, atmospheric pollution has posed a severe hazard to people’s health and production life [1,2], with PM2.5 serving as a major contaminant in many places [3]. In particular, in January 2013, the central–eastern region of China saw severe haze occurrences that caught the attention of all sectors of society [4]. Even though several measures have considerably contributed to the recent improvement in air quality, notably with the implementation of China’s Air Pollution Prevention and Control Action Plan in 2013 [5,6], many obstacles remain. Consequently, the study of PM2.5 and its influencing factors remains essential for PM2.5 mitigation and air quality improvement. Studying the spatial and temporal relationships between PM2.5 and socioeconomic factors facilitates sustainable economic development while mitigating PM2.5 pollution.

Numerous studies on PM2.5 have been conducted domestically and overseas due to rising public awareness about air pollution in recent years, primarily concentrated on pollution features, source analysis, standards and treatments for pollution control, and studies on influencing factors. For example, Chen et al. [7] disclosed the daily, monthly, and annual scales of PM2.5’s geographical variation pattern in China. Through the analysis and discussion of satellite remote sensing inversion data, Ma Zongwei [8] analyzed and discussed the variation characteristics, spatial distribution, and population exposure characteristics of PM2.5 concentrations in China. Zhang Zhipeng and Du Juan [9] and Liu Baoxian and Yang Wenyang [10] employed mass spectrometry and chemical mass reconstruction techniques to explore PM2.5 composition and sources in Guilin city during the summer and Beijing city in urban areas, respectively. As China’s socioeconomic development has accelerated in the 21st century, this has inevitably led to environmental issues like air pollution, which have increasingly attracted the attention of all facets of society. As a result, one of the major research topics in recent years has been the study of the factors that influence PM2.5, particularly socioeconomic factors [11,12].

There are numerous influencing factors for the study of PM2.5-related variables, principally natural and human influences. For instance, Nehzat [13] used a chemical mass balance model to develop a mathematical model for PM2.5 and PM10 data in the Sacramento region from 1991 to 1996. Results showed that the main factors influencing PM2.5 were seasonal vehicle emissions and smog emissions. Wang Shaojian et al. [14] examined the geographical heterogeneity of PM2.5 and its influencing factors in cities across the nation using a GWR model. However, only prefecture-level cities nationwide were included in previous studies of the variables impacting PM2.5 at the national municipal scale. Due to a lack of primary sources for socioeconomic statistics, studies rarely included several autonomous prefectures and regions in China. Most of these autonomous prefectures and regions are situated in the northwest and southwest; hence, they are geographically and socioeconomically very different from the eastern areas. As a result, there is a constraint in studies that examined the socioeconomic influences of PM2.5 at the national municipal level. Moreover, the spatial scale disparities in each influencing factor’s effects have rarely been taken into account in prior research, which has focused solely on the spatial heterogeneity of these socioeconomic influencing factors.

Currently, most researchers primarily use geographically weighted regression (GWR), for example, to analyze the spatial heterogeneity of regional influencing factors. However, establishing important characteristics such as bandwidth has received less focus, and the conclusions’ credibility is weakened by the incomplete consideration of the variations in the influencing variables’ scale of action. For this reason, certain limitations still exist. Although the multiscale geographically weighted regression (MGWR) model was developed later and had restricted applications, it has superseded GWR in studies during the past few years. Using the MGWR model, Wei et al. [15] analyzed the primary factors influencing changes in PM2.5 concentration in several regions of China and found that the dominant factors differed by region. In contrast to GWR, the MGWR model considers geographical scales when examining spatial heterogeneity, which improves the model in various ways and enables it to differentiate across scales of distinct variables. The MGWR model is one of the most cutting-edge methods for dealing with spatial heterogeneity. MGWR analysis of spatial data can better identify spatial heterogeneity and its spatial scale. Therefore, we tried to use the MGWR model to evaluate the spatial variability of PM2.5 socioeconomic impacts at different timepoints across the nation and the spatial size of each socioeconomic factor’s influence in this study.

Five socioeconomic indicators in all were chosen for this study. GDP per capita, the proportion of secondary industry, and population density are the more widely used and fundamental socioeconomic indicators and have been previously employed in the study of the relationship between PM2.5 and its influencing factors [16,17,18,19]. Industrial production has also been demonstrated to be closely related to air pollution [20,21]. Since data collection for many industry-related indicators is challenging, the number of industrial enterprise units above the scale was ultimately chosen to study the relationship between industry and PM2.5. Meanwhile, China’s economy has gradually transitioned in recent years from a stage of high-speed growth to a stage of high-quality development [22]. The proportion of general public budget revenues to GDP, which is a key indicator of the effectiveness of a nation or region’s economic development operations [23], is also one of the key reference indicators for the stage of high-quality development in the context of China’s economic optimization and transformation. Therefore, we selected the proportion of general public budget revenues to GDP as one of the indicators for analyzing the association between economic conditions and PM2.5.

In light of this, the paper selected China’s administrative divisions at the prefecture level as the fundamental unit. Annual average PM2.5 concentration and socioeconomic data were gathered for 2005, 2010, 2015, and 2020 for all municipalities, prefectures, autonomous prefectures, leagues, and regions in China, excluding Taiwan. Our main objectives were to (1) examine the spatial differentiation characteristics of socioeconomic factors and PM2.5 concentrations at various periods using spatial visualization and spatial autocorrelation methods, (2) analyze the association between global socioeconomic factors and PM2.5 from the perspectives of global and local variation features, and (3) explore the spatial scale of the influence of each explanatory variable on PM2.5 concentration, according to the MGWR model variable bandwidth and fitting results.

## 2. Materials and Methods

### 2.1. Data Sources

PM2.5 data in this study were obtained from the Atmospheric Composition Analysis Group (https://sites.wustl.edu/acag/datasets/surfacepm2-5/ (accessed on 18 June 2022)) at Washington University in St. Louis, MO, USA. The source data were raster data, converted into yearly PM2.5 mean data by comparing them to vector data of administrative regions in China. The essential socioeconomic data consisted of the gross domestic product (GDP), GDP per capita, the proportion of secondary industry value added to total GDP, the number of industrial enterprise units above the scale, the general public budget revenue of local finance, total population, population density, etc. Data were collected from the China Urban Statistical Yearbook, the China Regional Economic Statistical Yearbook, the China Ethnic Statistical Yearbook, and the relevant provincial (autonomous regions and municipalities directly under the Central Government), prefectural, and municipal statistical yearbooks and environmental statistical bulletins. Five indicators were acquired by additional collation and calculation: GDP per capita, the proportion of the secondary industry, the number of industrial enterprise units above the scale, the proportion of general public budget revenues to GDP, and population density. Due to limited access to data sources, socioeconomic indicator data for the Taiwan, Hong Kong, and Macao Special Administrative Regions, Sansha City in Hainan Province, and some autonomous counties in Xinjiang Uyghur Autonomous Region were not tallied. The final statistical research units consisted of 359 municipalities, autonomous prefectures, leagues, regions, and autonomous counties.

The variance inflation factor (VIF) was utilized for the test due to the possibility of bias in the results resulting from multicollinearity in each explanatory variable. A larger VIF value indicates greater multicollinearity, typically considered greater than 10. The variables were highly collinear; the variance and inflation factors were calculated as follows [24]:

(1) $$VIF=1-R_{i}^{2},$$

where $R_{i}$ is the negative correlation coefficient of the i-th independent variable $X_{i}$ for the remaining independent variables performing the regression analysis. Table 1 displays the test results for each explanatory variable from 2005 to 2020. The VIF values for each indication are less than 10, indicating that multicollinearity is not a concern in each explanatory variable.

### 2.2. Research Methods

#### 2.2.1. Spatial Autocorrelation

In this paper, global and local spatial autocorrelation were utilized to examine the evolution of spatial correlation of PM2.5 concentration values across the nation. Global spatial autocorrelation analyzes the spatial correlation and degree of spatial differences in a region from the perspective of the region. Moran’s I is a regularly used measure of global spatial autocorrelation that was first introduced by Moran [25]. To analyze the local spatial relationship (spatial clustering or spatial dispersion) of neighboring regions, a local indicator of spatial correlation (LISA) [26] can be selected. Local spatial autocorrelation can be used to quantify the spatial variability and importance between the yearly average PM2.5 concentration values in a particular region and those of the surrounding regions.

The following formulas are used to calculate global Moran’s I and local Moran’s I:

(2) $$I=\frac{n}{\sum_{i=1}^{n}\sum_{j=1}^{n}w_{ij}}\cdot \frac{\sum_{i=1}^{n}\sum_{j=1}^{n}w_{ij}\left(x_{i}-\overset{-}{x}\right)\left(x_{j}-\overset{-}{x}\right)}{\sum_{i=1}^{n}{\left(x_{i}-\overset{-}{x}\right)}^{2}},$$

(3) $$I_{i}=\frac{x_{i}-\overset{-}{x}}{\sigma^{2}}\sum_{j=1}^{n}w_{ij}\left(\frac{x_{j}-\overset{-}{x}}{\sigma }\right),$$

where I denotes global Moran’s I for the research area, $I_{i}$ denotes Moran’s I for city i, $x_{i}$ denotes the PM2.5 concentration in city i, and $x_{j}$ denotes the PM2.5 concentration in all other cities. Additionally, n denotes the total number of cities, and x is the mean PM2.5 concentration in China. The PM2.5 concentration’s standard deviation is $\overset{-}{x}$. The spatial weight matrix is called $w_{ij}$. I or $I_{i}$ values range from −1 to 1. Spatial autocorrelation in the cities is positive (negative) if I or $I_{i}$ values are positive (negative). The strength of the spatial correlation can be expressed by the value of I or $I_{i}$.

#### 2.2.2. Ordinary Least Squares

OLS (ordinary least squares) represents a simple global linear regression that focuses on creating a multivariate linear function between variables, calculated as follows:

(4) $$Y=\beta_{0}+\sum_{i=1}^{k}\beta_{i}x_{ij}+\varepsilon ,$$

where Y denotes the PM2.5 concentration, $\beta_{0}$ is the regression intercept, $x_{ij}$ denotes the explanatory variable, $\beta_{i}$ denotes the regression coefficient corresponding to the *i*-th explanatory variable, which can indicate the strength of the relationship between PM2.5 and socioeconomic indicators, and ε is the random error value. OLS regression models assume that the regression coefficients remain consistent across the spatial study area, ignoring the spatial nonstationarity of the variables and failing to reflect local variations in space [27].

#### 2.2.3. Multiscale Geographically Weighted Regression

The GWR model was first developed by Fortheringham [28]; it can tackle spatial autocorrelation and spatial non-stationarity problems that simple least squares cannot solve. Its basic form is as follows:

(5) $$y_{i}=\beta_{0}\left(u_{i},v_{i}\right)+\sum_{j-1}^{k}\beta_{j}\left(u_{i},v_{i}\right)x_{ij}+\varepsilon_{i},$$

where $y_{i}$ denotes the PM2.5 concentration in city *i*, $\beta_{0}\left(u_{i},v_{i}\right)$ denotes the constant term for city *i*, $x_{ij}$ denotes the explanatory variable, $\beta_{j}\left(u_{i},v_{i}\right)$ denotes the regression parameter of the independent variable at the data sampling point, and $\varepsilon_{i}$ denotes the stacking error term.

Unlike the conventional GWR model, the MGWR approach permits varying levels of spatial smoothing for each variable separately. MGWR generates a more realistic and practical spatial process model [29], constructed as follows [30,31]:

(6) $$y_{i}=\beta_{bw0}\left(u_{i},v_{i}\right)+\sum_{j=1}^{k}\beta_{bwj}\left(u_{i},v_{i}\right)x_{ij}+\varepsilon_{i},$$

where $y_{i}$ denotes the PM2.5 concentration in city *i*, $\left(u_{i},v_{i}\right)$ is the central coordinate at position *i*, and bwj represents the bandwidth used for the regression coefficient of the *j*-th variable, which is based on local regression, and the bandwidth has specificity, which is the most significant difference with GWR; $\beta_{bw0}\left(u_{i},v_{i}\right)$ and $\beta_{bwj}\left(u_{i},v_{i}\right)$ are the intercept and the regression coefficient of the *j*-th variable at *i*, respectively, and $\varepsilon_{i}$ is error term of the model at *i*. In this paper, the most commonly used quadratic kernel function and the AICc criterion are used, with the GWR estimate as the initial estimate, after which the difference between the actual value and the predicted value obtained from the initialized estimate is calculated, i.e., the initialized residual $\hat{\varepsilon }$.

(7) $$\hat{\varepsilon }=y-\sum_{j=1}^{k}{\hat{f}}_{j}.$$

This residual $\hat{\varepsilon }$ plus the first additive term ${\hat{f}}_{1}$ is regressed on the first independent variable $X_{1}$ in a classical GWR regression to find the optimal bandwidth $\beta_{w1}$, and a new column of parameter estimates ${\hat{f}}_{1}$ and $\hat{\varepsilon }$ to replace the previous estimates. The residuals are then added to a second additive term ${\hat{f}}_{2}$ with a second variable $X_{2}$, a second GWR regression is performed, and the parameter estimates for the second variable are updated. This is repeated until the last independent variable, $X_{k}$. The above is a complete cycle, repeated until the estimates converge to the convergence criterion [29]. This paper uses the convergence criterion for the difference between the current and the estimated regression coefficients calculated in the previous step.

## 3. Results and Discussion

### 3.1. Spatial and Temporal Features of Socioeconomic Variables and PM2.5 Concentrations in China

#### 3.1.1. Spatial and Temporal Distribution of Socioeconomic Influences across China

The socioeconomic data from 2005 and 2020 at the national municipal size were spatially shown in ArcGIS and divided into five levels using the natural or geometric interval approach. China’s islands in the South China Sea are depicted on the mini-map in the map’s lower right corner, along with the dashed line (the line of belonging to the islands in the South China Sea).

**Figure 1:**
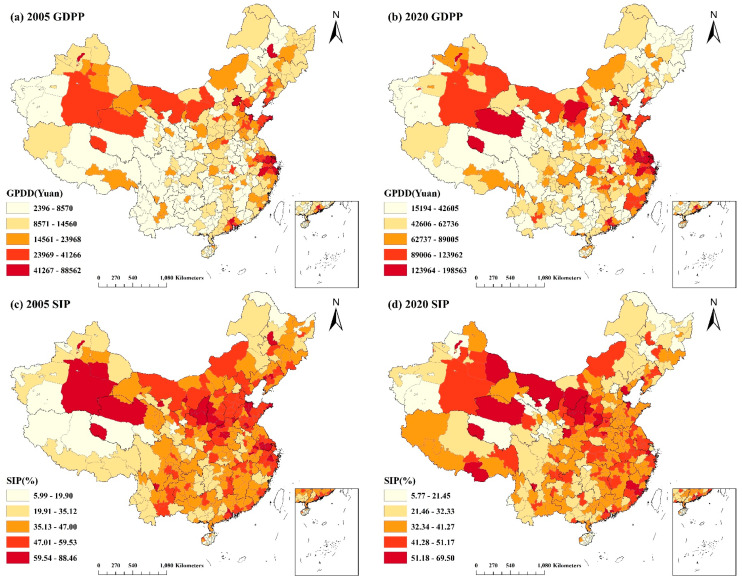

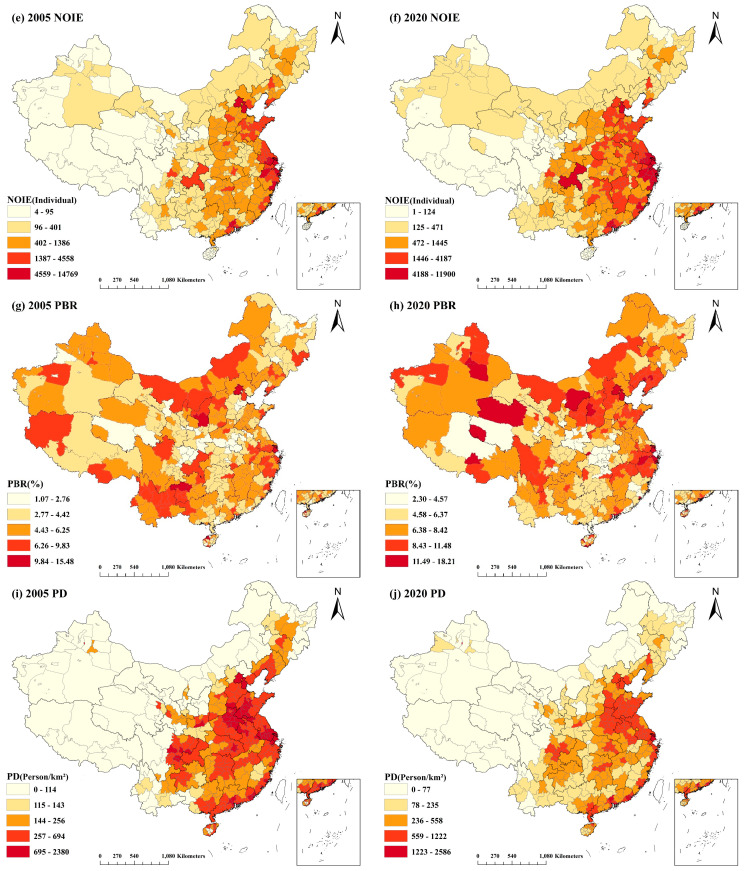
Spatial and temporal distribution of socioeconomic influences in China in 2005 and 2020: (**a**) spatial and temporal distribution of GDP per capita in 2005; (**b**) spatial and temporal distribution of GDP per capita in 2020; (**c**) spatial and temporal distribution of the proportion of secondary industry in 2005; (**d**) spatial and temporal distribution of the proportion of secondary industry in 2020; (**e**) spatial and temporal distribution of the number of industrial enterprise units above the scale in 2005; (**f**) spatial and temporal distribution of the number of industrial enterprise units above the scale in 2020; (**g**) spatial and temporal distribution of the proportion of general public budget revenues to GDP in 2005; (**h**) spatial and temporal distribution of the proportion of general public budget revenues to GDP in 2020; (**i**) spatial and temporal distribution of population density in 2005; (**j**) spatial and temporal distribution of population density in 2020.

Figure 1 shows that the GDPP increased significantly between 2015 and 2020. The GDPP was generally high in Beijing, Tianjin, Guangzhou, portions of the Yangtze River Delta, Shandong Province, and Fujian Province in the eastern coastal region, as well as in a few places in the northwest, but comparatively low in the southwest. The SIP was generally lower in 2020 than it was in 2005, with the highest and most consistent levels exceeding 50% in northern Shaanxi Province, Alashan League and Erdos City in Inner Mongolia Autonomous Region, Haixi Mongolian and Tibetan Autonomous Prefecture in Qinghai Province, and Bayingoleng Mongol Autonomous Prefecture, Turpan City, and Hami City in Xinjiang Uyghur Autonomous Region. The Yangtze River Delta region, Shandong Province, and Hebei Provinces were other regions with a comparatively large number of secondary industries. The NOIE varied significantly across the country. It was highly concentrated in the east, with the highest concentration in the Beijing–Tianjin–Hebei region, as well as Shandong, Jiangsu, and Zhejiang Provinces. Nevertheless, the NOIE in 2020 was lower than in 2005. Moreover, the NOIE in the western area, particularly in the Tibet Autonomous Region and Qinghai Province, could have been much higher. In 2020, it was also evident that the NOIE in most regions in the northwest was expanded. As with GDPP, PBR was likewise on the rise. In economically developed regions such as Beijing, Tianjin, Shanghai, and Jiangsu province, as well as in northern Shaanxi Province, sections of southwest China, and northern Xinjiang Uyghur Autonomous Region, the PBR was relatively high. In 2015 and 2020, the PD of the eastern and western regions varied significantly. In the northwest and southwest regions, along with the majority of Heilongjiang Province, the PD was extremely low, and the trend was negligible. The PD was rising in the east, especially in the Beijing–Tianjin–Hebei region, Shandong, Henan, and Jiangsu Provinces, and the coastal regions.

#### 3.1.2. Spatial and Temporal Distribution of PM2.5 Concentrations in China

**Figure 2:**
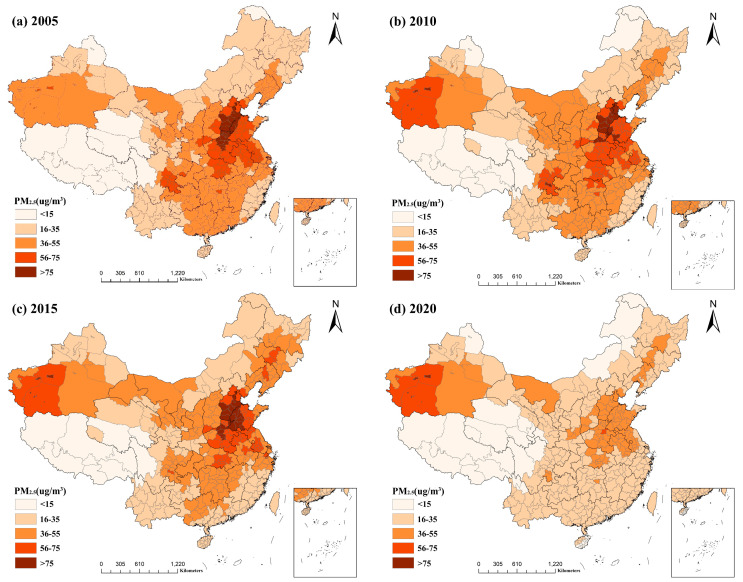
Spatial and temporal distribution of national PM2.5 concentrations from 2005 to 2020: (**a**) spatial and temporal distribution of PM2.5 concentrations in 2005; (**b**) spatial and temporal distribution of PM2.5 concentrations in 2010; (**c**) spatial and temporal distribution of PM2.5 concentrations in 2015; (**d**) spatial and temporal distribution of PM2.5 concentrations in 2020.

The yearly average concentration values were categorized according to the Ambient Air Quality Standard (GB3095-2012), divided into five classes: <15 µg/m^3^, 16–35 µg/m^3^, 36–55 µg/m^3^, 56–75 µg/m^3^, and >75 µg/m^3^. The results are depicted in Figure 2, which demonstrates that the national PM2.5 concentrations increased slightly in 2010 compared to 2005 but reduced in 2015 and 2020, with the most substantial decrease occurring in 2020 when most locations fell below 35 µg/m^3^. This suggests a general downward trend in the annual average PM2.5 concentration over the past few years, which is likely attributable to the implementation of the "Ten Atmospheric Articles" of China since 2013 and the active measures taken by local governments to introduce air pollution prevention plans and policies one after the other [32]. Moreover, with the implementation of the new development idea and the accelerated transformation of old and new dynamics during the past few years, air quality has improved in the majority of the country’s regions [33].

According to the Ambient Air Quality Standard (GB3095-2012), 15 µg/m^3^ and 35 µg/m^3^ are the annual average primary and secondary concentration limits of PM2.5 in China, respectively [3]. The combined spatial distribution of PM2.5 data from 2005 to 2020 revealed considerable regional disparities. The majority of areas with annual average PM2.5 concentrations below 35 µg/m^3^ were located in economically underdeveloped plateau and mountainous regions, such as the Tibet Autonomous Region in the west and southwest of China, the western part of Sichuan Province, and the Qinghai and Yunnan Provinces, as well as the northern part of Xinjiang Uyghur Autonomous Region, the northern part of the Inner Mongolia Plateau, northeastern Heilongjiang Province, and Hainan and Taiwan provinces. Among them, Tibet is a vast and sparsely populated country with little human activity, which has the lowest annual average PM2.5 concentration, remaining below 15 µg/m^3^. High PM2.5 concentrations were predominantly found in the Beijing–Tianjin–Hebei region, Shandong, Henan, Hubei, and Jiangsu Provinces, and portions of eastern Sichuan Province. These locations are primarily located in the core regions of China, which are characterized by a dense population, developed industry, and rapid socioeconomic development; thus, PM2.5 concentrations are high and concentrated. In addition, PM2.5 pollution was more severe in the Hotan, Kashgar, and Aksu regions of the southwestern Xinjiang Uyghur Autonomous Region. The high wind and dusty desert areas in the Taklamakan Desert region of Xinjiang primarily increase the number of fine particles in the air, thereby increasing PM2.5 concentrations [34].

#### 3.1.3. Analysis of the Global Spatial Autocorrelation of the National PM2.5 Concentration

The software GeoDa was utilized to create the spatial weight matrix and calculate the global Moran’s I for the spatial distribution of PM2.5 concentrations in China for four time periods spanning 2005 to 2020.

As shown in Table 2, global Moran’s I values for PM2.5 concentrations were all positive, except for the year 2020, when the values were significantly lower and were over 0.8 most of the time, with a p-value of 0.001, which was highly significant. The z-values representing standard deviations were significant and positive, with no notable variation across years. This suggests a considerable spatial positive correlation, spatial homogeneity, and spatial clustering of PM2.5 at the municipal scale in China. In the table, E[I] represents the expected value of I, and the result was −0.003 throughout all four time periods. The standard deviation of I is denoted by SD; all values were close to 0.034. According to the results of these two indicators, the positive PM2.5 pollution spillover effect in China is reasonably consistent and at a high level.

#### 3.1.4. Local Spatial Autocorrelation Analysis of National PM2.5 Concentrations

The spatial statistics tool in ArcGIS was used to perform clustering and outlier analysis to generate spatial clustering maps of PM2.5 for four time periods between 2005 and 2020 to find areas with significant spatial correlation and different types of geographical clustering of PM2.5.The clusters were categorized as “high–high” (HH), “low–low” (LL), “high–low” (HL), “low–high” (LH), and “not significant”.

**Figure 3:**
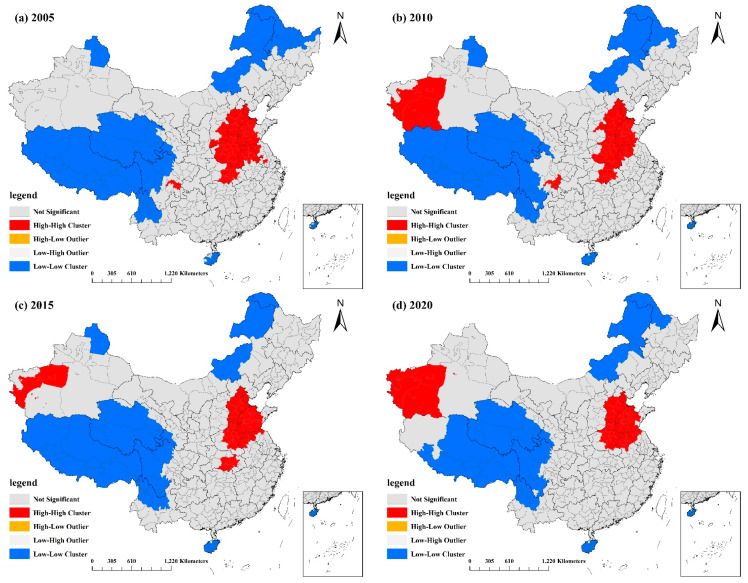
Spatial clustering of annual average PM2.5 concentration values in China from 2005 to 2020: (**a**) spatial clustering of annual average PM2.5 concentration values in 2005; (**b**) spatial clustering of annual average PM2.5 concentration values in 2010; (**c**) spatial clustering of annual average PM2.5 concentration values in 2015; (**d**) spatial clustering of annual average PM2.5 concentration values in 2020.

The outcomes are depicted in Figure 3. The HH and LL forms of PM2.5 in China were heavily concentrated, and there was no notable change in their spatial distribution across the country between years. Beijing, Tianjin, Hebei Province, Henan Province, central and western Shandong Province, northern Jiangxi Province, central Hubei Province, and a tiny portion of Shanxi Province and Jiangsu Province comprised the HH agglomeration areas. This is significantly related to the region’s dense population, established economy, and high level of urbanization development [35], particularly in Shandong, Hebei, and Henan Provinces, where there is a large population, a high total energy consumption, and a large fraction of industry. In addition, the Hotan, Kashgar, and Aksu regions in the western part of the Xinjiang Uyghur Autonomous Region, next to the Taklamakan Desert, exhibited a "high–high" concentration due to their windy and sandy local environment and climate. LL PM2.5 aggregation was most prevalent in the Tibet Autonomous Region, Qinghai Province, northwestern Yunnan Province, Ganzi Tibetan Autonomous Prefecture in western Sichuan Province, northeastern Inner Mongolia Autonomous Region, Daxinganling and Heihe in northwestern Heilongjiang Province, and the Altai region of Xinjiang. These locations are less densely populated, are less economically developed, and have low human activity intensity [34], resulting in light PM2.5 pollution.

### 3.2. Spatial Regression Results of the Relationship between Socioeconomic Factors and PM2.5 in China

#### 3.2.1. Global Spatial Regression Analysis—OLS Model Results

From 2005 to 2020, GeoDa software was used to estimate OLS regression models for 359 prefecture-level and higher cities in China, excluding Taiwan Province, Hong Kong, and Macau. The regression and fit results for each variable of the developed OLS model are presented in Table 3 and Table 4. Most socioeconomic indicators were strongly linked with PM2.5 and passed the significance test. Similarly, the F-statistic values ranged from 13.755 to 64.733, all of which remained significant at the 1% level, showing a linear relationship between the explanatory and dependent variables. Most periods exhibit a negative correlation between GDPP and PM2.5, with the strongest association occurring in 2005, while the influence of GDPP was reduced in other years. In 2020, however, the impact of the pandemic of coronavirus disease 2019 (COVID-19) caused human activity and many industries to come to a standstill and the economy to suffer a major shock due to the strict containment and prevention measures implemented within China. Meanwhile, the air quality has significantly improved as a result of strict control measures [36]. Thus, there was a slight positive association between GDPP and PM2.5. The SIP and PD exhibited significant positive effects in all four time periods, demonstrating that these two variables strongly affect PM2.5 pollution. In contrast, the NOIE had no significant effect on PM2.5 during the 2005 and 2010 periods. However, the regression coefficients of NOIE grew dramatically in 2015 and 2020, exhibiting a much stronger positive association and negative correlation, respectively. The PBR was significantly adversely connected with PM2.5 in the 3 years, except for 2020, where the effect was insignificant.

In conjunction with the model fit results in Table 4, it was determined that the adjusted R^2^ values ranged from 0.160 to 0.478, which are not exceptionally high; in 2020, both R^2^ and adjusted R^2^ values were below 0.2, which is a significant deviation from other years, and the AIC values were greater between 2810.6 and 2938.9. This shows that, despite the significance of the regression coefficients for the majority of the indicators, they need to explain the situation adequately. At the 1% significance level, the findings of the Jarque–Bera test indicated that the residuals varied from the normal theoretical distribution, while a study of the spatial autocorrelation of the residuals to calculate the Moran’s I revealed a significant spatial autocorrelation. The findings of the BP test were likewise significant at the 1% level, showing heteroskedasticity-induced bias in the standard errors. The initial results imply that the OLS model statistics were biased, the fit was not optimal, and the analytical process had limits. Consequently, additional analysis was required utilizing local spatial regression models such as GWR and MGWR.

#### 3.2.2. Local Spatial Regression Analysis—GWR and MGWR Model Results

This research used MGWR 2.2 software to perform GWR and MGWR with PM2.5 concentrations as the dependent variable and the five explanatory factors to obtain regression findings for four time periods from 2005 to 2020. As shown in Table 5, the model’s R^2^ and adjusted R^2^ values for each year were much higher than those of the OLS model. The AICc values were significantly lower, demonstrating that the GWR and MGWR models provided more accurate predictions than the OLS model. The MGWR model had higher adjusted R^2^ values, smaller AICc values, and residual sums of squares than the traditional GWR model. Consequently, the MGWR model was the most suitable.

**Figure 4:**
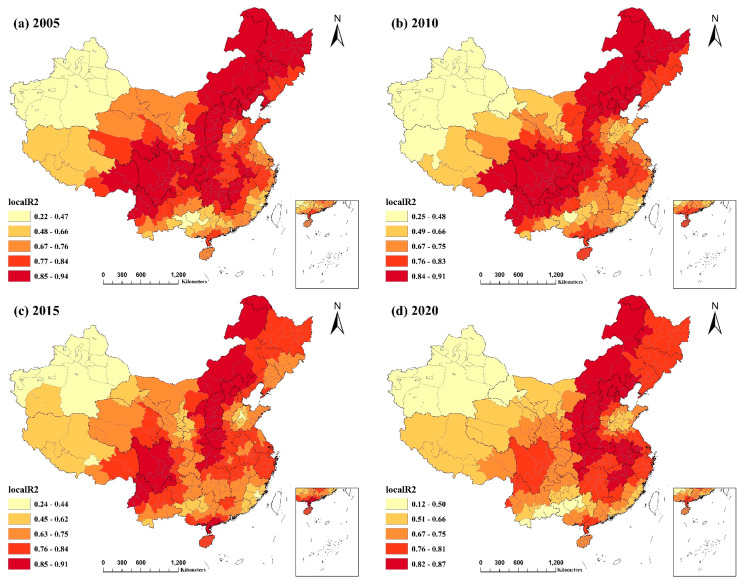
Spatial distribution of local R^2^ values for the GWR model from 2005−2020: (**a**) spatial distribution of the local R^2^ values of the GWR model for 2005; (**b**) spatial distribution of the local R^2^ values of the GWR model for 2010; (**c**) spatial distribution of the local R^2^ values of the GWR model for 2015; (**d**) spatial distribution of the local R^2^ values of the GWR model for 2020.

**Figure 5:**
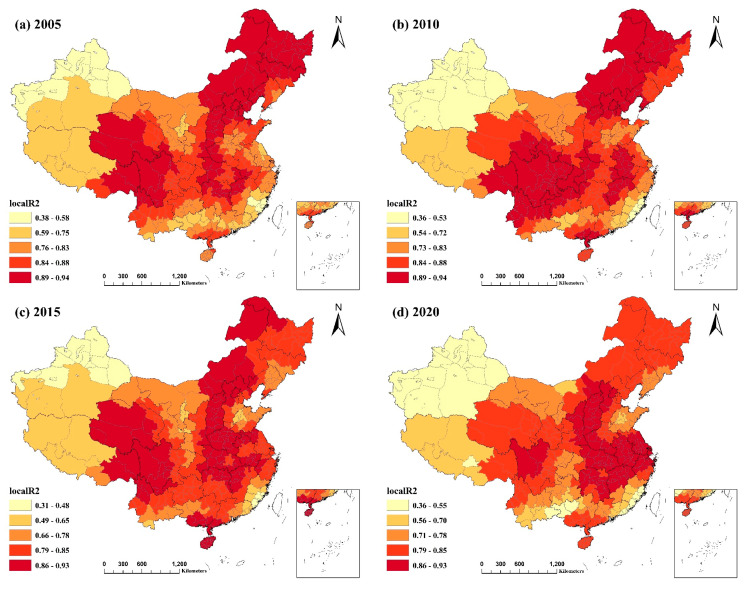
Spatial distribution of local R^2^ values for the MGWR model from 2005−2020: (**a**) spatial distribution of the local R^2^ values of the MGWR model for 2005; (**b**) spatial distribution of the local R^2^ values of the MGWR model for 2010; (**c**) spatial distribution of the local R^2^ values of the MGWR model for 2015; (**d**) spatial distribution of the local R^2^ values of the MGWR model for 2020.

Figure 4 and Figure 5 depict the spatial distribution of the local R^2^ for the GWR and MGWR models, respectively. Local R^2^ values for both models surpassed 0.5, except for Xinjiang Uygur Autonomous Region and a few other regions, while the values for more than half of the regions exceeded 0.7. The local R^2^ values for MGWR were generally higher than those for GWR. This demonstrates that the MGWR model was a better fit and that the indicators provided a better explanation for PM2.5 concentrations. For the four selected years, the spatial distribution of the local R^2^ values for both models was comparable and did not vary considerably across years. Northeast China, several provinces in East China, northern and eastern Inner Mongolia Autonomous Region, Beijing–Tianjin–Hebei region, northern Yunnan Province, Shanxi, Shaanxi, and Sichuan Provinces, and a few locations in Guizhou Province had the highest local R^2^ values. This suggests that the combined explanatory power of the identified factors impacting PM2.5 concentrations was more significant in the majority of cities in these regions. In contrast, the R^2^ values were often modest in most regions of Xinjiang and Tibet, particularly Xinjiang. This result may be due to the low intensity of human activity in these locations, which are less affected by socioeconomic influences and more by local natural characteristics such as climate, terrain, and geography, resulting in a poorer model fit.

### 3.3. Analysis of the Spatial Heterogeneity of Socioeconomic Factors and Their Spatial and Temporal Evolution in China

As the MGWR model provided the best fit, this article geographically visualized the regression coefficients of each explanatory variable in the MGWR model using ArcGIS. It then analyzed the spatial differences in the role and intensity of socioeconomic influencing factors.

#### 3.3.1. GDP Per Capita

**Figure 6:**
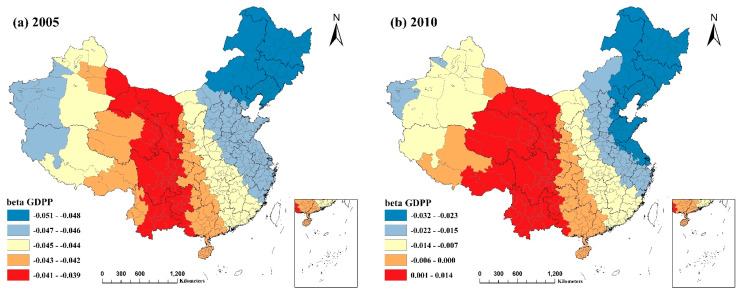

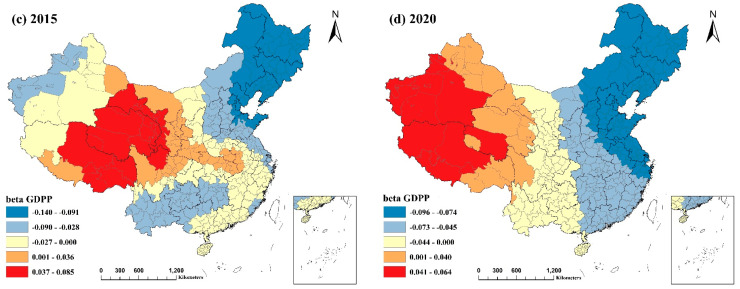
Spatial and temporal distribution of regression coefficients of GDP per capita in the MGWR model, 2005−2020: (**a**) spatial and temporal distribution of regression coefficients for GDP per capita in 2005; (**b**) spatial and temporal distribution of regression coefficients for GDP per capita in 2010; (**c**) spatial and temporal distribution of regression coefficients for GDP per capita in 2015; (**d**) spatial and temporal distribution of regression coefficients for GDP per capita in 2020.

As shown in Figure 6, the association between GDPP and PM2.5 was negative in most regions, particularly in the east, indicating that a rise in GDPP would lower PM2.5 pollution in most regions of China. APpeople’s awareness of environmental protection has increased, science and technology have advanced, local economies’ industrial structure has been optimized, and a new economic development model of green recycling has been sought [37]. Accordingly, many places, mainly economically developed regions, have achieved gradual harmony between economic and environmental development. Greater absolute values of the regression coefficients were primarily observed in the northeastern region, the northeastern portion of the Inner Mongolia Autonomous Region, and certain parts of the eastern coast, indicating that the negative effect of GDPP on PM2.5 was more pronounced in these regions.

Nevertheless, beginning in 2010, certain regions in the northwest and southwest of China exhibited a positive correlation between GDPP and PM2.5. In 2020, the positively correlated regions were concentrated in the Tibet Autonomous Region, the Xinjiang Uyghur Autonomous Region, Qinghai Province, and Gansu Province in northwest China. On the one hand, the scant vegetation, high sandiness, and peaceful natural environment of the northwest rendered them susceptible to air pollution. On the other hand, due to the generally poor level of economic development, lagging economic patterns and public services [38], low levels of resource utilization in these regions, and the gradual shift of highly polluting industries from economically developed eastern regions to the backward regions over the past few years, there may have been a phenomenon of development at the expense of the environment.

#### 3.3.2. The Proportion of Secondary Industry

**Figure 7:**
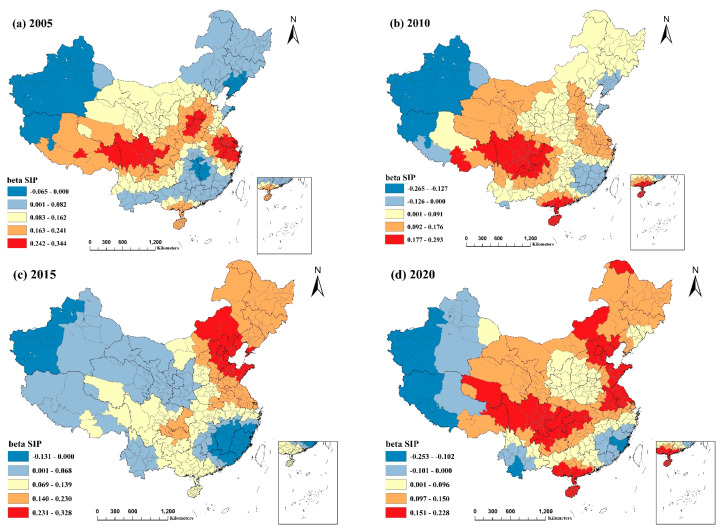
Spatial and temporal distribution of regression coefficients of the proportion of the secondary industry in the MGWR model, 2005−2020: (**a**) spatial and temporal distribution of regression coefficients for the proportion of the secondary industry in 2005; (**b**) spatial and temporal distribution of regression coefficients for the proportion of the secondary industry in 2010; (**c**) spatial and temporal distribution of regression coefficients for the proportion of the secondary industry in 2015; (**d**) spatial and temporal distribution of regression coefficients for the proportion of the secondary industry in 2020.

As depicted in Figure 7, the regression coefficients for SIP in most regions were positive. They had large values, indicating that SIP positively correlated with PM2.5 and that reducing SIP was conducive to reducing PM2.5 pollution. This is also consistent with the results of other investigations [18,19,39]. The value of the regression coefficient in 2020 was lower, which may be attributable to the implementation of air pollution prevention and control actions since 2014. Additionally, measures such as modifying and upgrading the industrial structure have gradually reduced the impact of the secondary industry’s share on PM2.5 pollution [40].

The positive effect of SIP on PM2.5 pollution was most significant in the Beijing–Tianjin–Hebei region and parts of Shandong and Henan provinces in northern China, the Yangtze River Delta region, southern Guangdong and Guangxi provinces, Hainan province in southern China, and Sichuan and Chongqing cities in southwestern China, where the coefficient values were the highest. The reason may be that the proportion of highly polluting and energy-consuming secondary industries is relatively high in these regions, particularly in traditional industrial zones such as the Beijing Tianjin Tangshan Industrial Zone and Shanghai Nanjing Hangzhou Industrial Zone. Furthermore, some traditional coastal industrial zones, with large populations, high demand and consumption of energy and resources, low energy utilization rates, and irrational industrial structures, emit more pollutants into the air due to their inefficient design [41]. In regions of Xinjiang and Tibet, where a negative correlation was observed, local natural elements may be more significant than the production value of the secondary industry, which is, therefore, not a significant determinant. In addition, Fujian and Jiangxi provinces in southern China showed a negative correlation. The reason is that the agglomeration effect is favorable to increasing efficiency and decreasing air pollution. After years of economic and industrial reorganization, new technologies, enhanced science and technology, and energy efficiency have fostered coordinated and steady local economic growth while reducing air pollution emissions [42,43].

#### 3.3.3. The Number of Industrial Enterprises above the Scale

**Figure 8:**
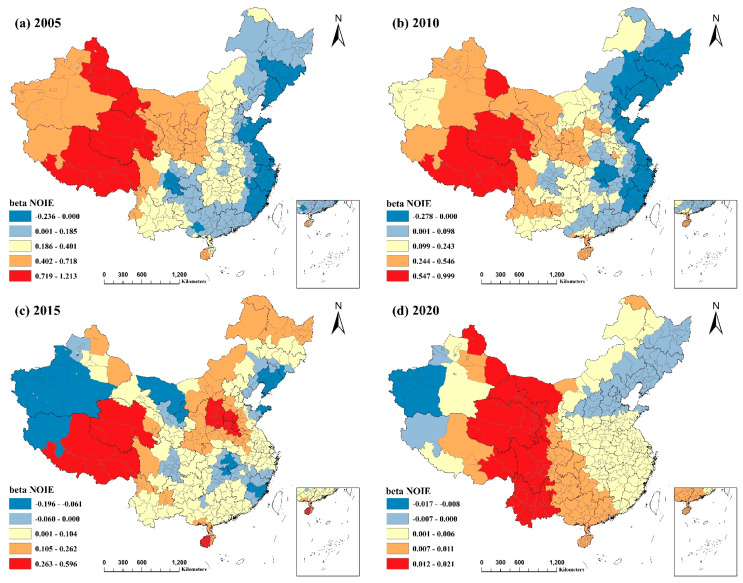
Spatial and temporal distribution of regression coefficients of the number of industrial enterprises above the scale in the MGWR model, 2005−2020: (**a**) spatial and temporal distribution of regression coefficients for the number of industrial enterprises above the scale in 2005; (**b**) spatial and temporal distribution of regression coefficients for the number of industrial enterprises above the scale in 2010; (**c**) spatial and temporal distribution of regression coefficients for the number of industrial enterprises above the scale in 2015; (**d**) spatial and temporal distribution of regression coefficients for the number of industrial enterprises above the scale in 2020.

As shown in Figure 8, the association between NOIE and PM2.5 was predominantly positive. However, the effect was not statistically significant in the eastern regions, and certain regions exhibited a negative correlation. From 2005 to 2020, the absolute value of the regression coefficient for NOIE tended to decline in an increasing number of regions, with the trend being most pronounced in 2020. This result suggests that NOIE was considerably and positively connected with PM2.5 but that the effect on PM2.5 was steadily diminishing, both positively and negatively, with a minor effect occurring in 2020. This phenomenon takes into account the possibilities related to the recently promoted green industrial transition and the impact of COVID-19 2020 on industrial companies.

According to the graph, the areas where PM2.5 was positively correlated with NOIE in 2005 and 2010 were primarily found in Northwest China and the Tibet Autonomous Region. This phenomenon may be attributable to the fact that most industrial enterprises in the region are highly polluting and energy-intensive. The only regions with a negative correlation were the Northeast and portions of the East Coast. The reason may be due to the region’s flat terrain, proximity to the ocean, significant monsoon effect, and high average annual wind speed, which provide favorable conditions for the dispersion of air pollutants emitted by industrial enterprises as a result of industrial activities [44]. At the same time, meteorological factors such as precipitation also influence the results. In contrast, in 2015 and 2020, the regions where NOIE was negatively correlated with PM2.5 were primarily located in the Aksu, Hotan, and Kashgar regions of the southern Xinjiang Uygur Autonomous Region, as well as the Ali region of the Tibet Autonomous Region, although not significantly. As these locations are sparsely inhabited [45], the number of industrial companies is low, and the model fit could be better; hence, the obtained results need to explain the issue adequately.

#### 3.3.4. The General Public Budget Revenue as a Proportion of GDP

General public budget income is a vital indicator of a country’s or region’s financial strength. The general public budget revenue as a proportion of GDP is a vital indicator of the quality of economic development activities [23]. From 2005 to 2020, Figure 9 demonstrates that PBR negatively influenced PM2.5. In 2005, the absolute value of the coefficient was more remarkable, and the impact was most pronounced. However, the influence in the three periods from 2010 to 2020 was less pronounced and remained relatively stable.

When the data of the four timepoints were combined, it was discovered that the negative influence of PBR on PM2.5 diminished from north to south. In Northeast China, sections of Inner Mongolia, the Beijing–Tianjin–Hebei region, Shandong Province, Shanxi Province, Xinjiang Uyghur Autonomous Region, and the Ali region in Tibet, PM2.5 was most affected by PBR. The reason may be that these regions have relatively high PM2.5 concentrations and heavier air pollution, as well as the fact that many regions still have an inappropriate energy structure and industrial layout, and that the quality of economic development in these regions is lower than in the south. Therefore, in these places, the increase in the proportion of general public budget revenues, i.e., the enhancement of economic performance, had a more vital role in reducing PM2.5 pollution.

#### 3.3.5. Population Density

**Figure 10:**
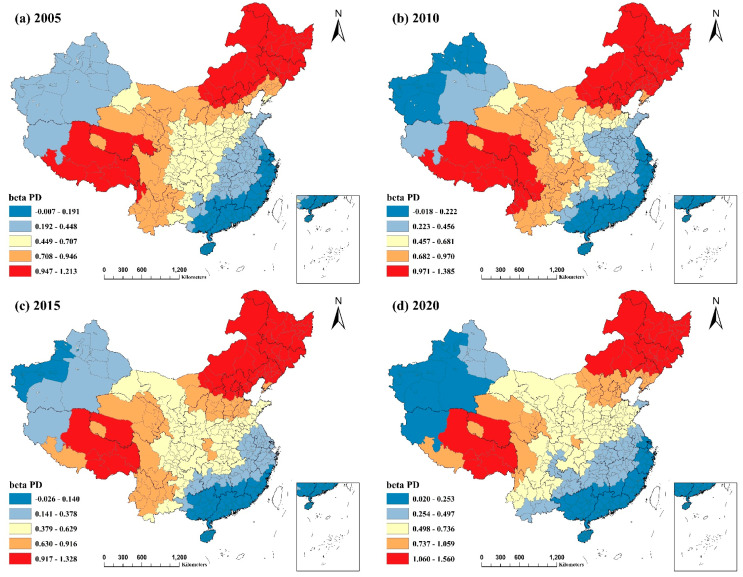
Spatial and temporal distribution of regression coefficients of population density in the MGWR model, 2005−2020: (**a**) spatial and temporal distribution of regression coefficients for population density in 2005; (**b**) spatial and temporal distribution of regression coefficients for population density in 2010; (**c**) spatial and temporal distribution of regression coefficients for population density in 2015; (**d**) spatial and temporal distribution of regression coefficients for population density in 2020.

Figure 10 demonstrates that PD had a considerable positive connection with PM2.5, and its effect tended to be significantly stronger. Higher population densities tend to lead to higher energy consumption, increased pollutant emissions [46], and traffic congestion caused by population concentrations, which is not conducive to the complete combustion of vehicle fuels [47]. The decreased efficacy of air dispersion in densely populated areas with tall buildings results in PM2.5 accumulation [48]. These studies showed that heavily inhabited regions are more prone to air pollution. Mainly, the positive effect of PD on PM2.5 pollution was most pronounced in the figure’s northeastern parts of China and the Tibet Autonomous Region, Qinghai Province, and Yunnan Province. PD statistics reveal that these regions, such as Inner Mongolia Autonomous Region, Qinghai Province, Tibet Autonomous Region, and western Sichuan Province, have low PM2.5 concentrations and low population densities and, therefore, do not cause severe air pollution or population problems. Combined with a more backward economic development and low degree of air management, PD significantly impacts PM2.5 during the development process.

In contrast, the more economically developed eastern regions, particularly the southeastern coastal districts, had a more significant PD and intensity of human activity. However, the influence of PD on PM2.5 pollution was weaker compared to other locations. The reason may be due to the influence of population agglomeration on PM2.5, i.e., population agglomeration enhances resource sharing and public transportation efficiency [22]. Along with the rising knowledge of environmental protection and environmental demands, as well as the emphasis that society places on pollution prevention and control, the government is taking active steps to enhance air quality, thereby diminishing the effect of human activities on PM2.5.

### 3.4. Spatial Scale Analysis of the Role of Socioeconomic Factors on PM2.5

As shown in Table 6, the classical GWR could only represent the average of the action scales of the individual explanatory variables, with bandwidths ranging from 54 to 70 over the four time periods, which did not vary significantly overall. In contrast, the MGWR could reflect the differential scales of action of various explanatory variables, with larger scales indicating less spatial heterogeneity in the effects of the influencing factor and vice versa [49]. It can be seen from the table that the bandwidth of different variables in the MGWR model varied considerably, indicating that different socioeconomic factors had varying scale effects.

The results in the table show that the bandwidth values of GDPP were all high, reaching over 329 under most periods, which is equal to the total sample, except in 2015 when the bandwidth value was 135, but still relatively large, indicating that there was almost no spatial heterogeneity. Furthermore, from the results in Figure 3, Figure 4, Figure 5 and Figure 6, it can be found that the absolute values of the regression coefficients were minor and did not differ significantly across the country, all of which indicate that the effect of GDPP on PM2.5 was broadly consistent across the country. The scale effect of PBR was small in 2005, with a bandwidth of 53, and then increased significantly from 2010 to 2020, before gradually became a global variable, with a maximum of 353 in 2020. The spatial heterogeneity of the effect was small and decreased after 2010, which is also consistent with the range and distribution of the coefficients in the regression coefficient results.

The scale effect for the SIP and PD was negligible in all periods, as well as for the NOIE in all three time periods from 2005 to 2015. The bandwidths values for NOIE and PD ranged from 43 to 46. In contrast, the scale effect of SIP was slightly minimal, with a bandwidth ranging from 53 to 86. This result indicates that the spatial heterogeneity of the effect of these influencing factors was more remarkable. The spatial heterogeneity of the effects of these factors was evident from the spatial heterogeneity of the socioeconomic factors influencing PM2.5 in the preceding section, which demonstrated significant differences between regions. In contrast, in 2020, the bandwidth value of NOIE reached 353, and the scale effect became large, as indicated by the regression coefficient. This change may be since, on the one hand, in recent years, especially since the introduction of the transformation of the old and new dynamics, promoting the transformation of green industrial growth has become essential support for China’s high-quality economic development and a key to achieving peak carbon and carbon neutrality. Many regions have begun to change their past industrial development model, which relied on resource inputs, high energy consumption, and high carbon emissions [50,51]. The NOIE is on the decline. On the other hand, COVID-19 in 2020 had a significant impact on China’s industry in the short term and to varying degrees on different industrial sectors and regions [52], with some industrial enterprises experiencing shutdowns, a slow recovery of production capacity, and closures. These elements diminished PM2.5’s regional variability and spatial heterogeneity of effects, as well as the influence of industrial influences such as NOIE.

### 3.5. Recommendations and Policy Responses

Air pollution and its control are still a hot topic in today’s society. In order to effectively reverse the trend of PM2.5 pollution, it is essential to pinpoint the underlying reasons, implement practical policies and actions that take into account local circumstances, and support the harmonious growth of the economy and environment. The following recommendations and policy responses are made in response to this study’s experimental findings about PM2.5 pollution:

(1) Differentiated zoning to put control policies into effect. China is a huge country, and, due to regional disparities in economic development and environmental conditions, the level of PM2.5 pollution varies from region to region. Additionally, there is significant spatial heterogeneity in the connection between PM2.5 and socioeconomic factors. Therefore, it is essential to create a governance strategy that is ideal for regional development while maintaining some concentration, as well as to stop pollution leakage between various regions. At present, the Beijing–Tianjin–Hebei region, Shandong province, and Henan province in the north are still the core areas of governance.

(2) Joint prevention and control. China’s reaction to environmental pollution, especially the prevention of air pollution, is currently centered on regional "joint prevention and control". Inter-regional collaboration and the development of a successful joint prevention and control mechanism are necessary for the management of PM2.5 pollution. Prior to developing unified environmental standards and laws, it is crucial to identify the parties responsible for different types of pollution in each region, especially for areas with serious air pollution. To develop a long-term mechanism for regional cooperation in the fight against haze, a regional PM2.5 monitoring platform should be established to share environmental information, collaborate with one another, and converge environmental rules.

(3) Improve the economic structure and pursue a new, high-quality, green economic development model. Although the impact is not obvious, GDP per capita still has a positive effect on reducing PM2.5 pollution. Therefore, it will be important to maintain the promotion of both sound environmental protection and economic development that is sustainable. At the same time, in order to encourage high-quality economic development, the general public budget revenue as a proportion of GDP should be raised, e.g., accelerating the development of the tertiary sector, particularly contemporary service industries, and enhancing the creation of pillar industries, as well as strengthening revenue collection and management, improving the content of assessments, supporting scientific and technological innovation, and improving the quality of tax sources [53]. These actions have a more blatantly beneficial impact on lowering PM2.5 concentration.

(4) Strengthen pollution control. The primary sources of air pollution emissions are secondary industry and industrial enterprise concentration. On the one hand, the industrial structure needs to be continuously optimized, the proportion of secondary industries needs to be decreased, and the development of high-tech and environmentally friendly industries needs to be vigorously promoted in order to further reduce air pollution, particularly in heavy industrial cities in the north. On the other hand, in order to reduce air pollution, the growth of a new economy should be accompanied by the abolition of highly energy-intensive and heavily polluting businesses, a steady decline in the number of outdated industrial businesses, and the encouragement of green industrial transformation.

(5) Create a practical plan for population distribution to direct population diversion. Future urban development should specifically avoid the blind expansion of urban land, and the relationship between urban expansion and population size should be coordinated to encourage population movement from the central city to the surrounding areas, as well as the transition from single-center to multi-center urban development. At the same time, rational planning and scientific land use should be carried out to alleviate the conflict between people and land, and targeted initiatives should be actively taken to deal with the social and environmental problems caused by population.

## 4. Conclusions

This paper utilized PM2.5 and socioeconomic data from 2005 to 2020. The spatiotemporal characteristics of PM2.5 and socioeconomic factors and the spatiotemporal relationships among them were analyzed through spatial visualization, spatial autocorrelation, global spatial regression analysis (OLS), and local spatial regression analysis (MGWR) methods. The research results obtained are as follows.

(1) GDP per capita, general public budget revenue as a proportion of GDP, and population density all typically tended to rise between 2005 and 2020 and were all concentrated in the eastern provinces. The proportion of secondary industry decreased, and the eastern sea areas had the majority of the higher concentrations of industrial companies above the scale, but the number in the eastern regions decreased. At the same time, the national PM2.5 concentration tended to decline generally, and the spatial distribution had a sizable positive spatial correlation. PM2.5 concentrations were high, and there was a "high–high" concentration in densely inhabited and economically developed places such as Beijing, Tianjin, Hebei, Shandong, and Henan provinces, as well as in the windy and sandy areas of Xinjiang. On the other hand, there was less PM2.5 pollution and little clustering in regions with less intense human activity and less economic development, such as Tibet.

(2) From the findings of the global spatial regression analysis using the OLS model, we discovered that PM2.5 was mostly adversely connected with general public budget revenue as a proportion of GDP, and significantly positively correlated with the proportion of secondary industry and population density. The regional variability of the influence of socioeconomic factors on PM2.5 was further examined using the MGWR model. Since 2010, The share of general public budget revenue in GDP had a significant negative effect on PM2.5 in all regions. GDP per capita, on the other hand, had a weak impact on PM2.5 reduction, although it had a positive effect. Furthermore, in recent years, in some provinces in the northwest and southwest, such as Gansu, Qinghai, and Sichuan, as well as in some areas of Tibet and Xinjiang, GDP per capita showed a weaker positive correlation with PM2.5. In addition, there were positive relationships between PM2.5, the proportion of secondary industry, the number of industrial enterprises above scale, and population density in most regions. Additionally, the number of industrial enterprises above scale generally had a weaker effect on PM2.5 in 2020 than in previous years, except in some provinces on the eastern coast and in some areas of Xinjiang, where it had a negative effect on PM2.5.

(3) Different socioeconomic factors contributed to PM2.5 at different spatial scales. The global variable with the broadest scope of action was GDP per capita, followed by the general public budget revenue as a proportion of GDP, which was low in 2005 but rapidly rose in successive years. The proportion of secondary industry played a relatively minor role, and the scales of action for population density and the number of industrial enterprises above the scale were the most constrained. However, in 2020, the spatial scale of the contribution of the number of industrial enterprises above the scale to PM2.5 was very large, very much unlike previous years.

## Figures and Tables

**Figure 9:**
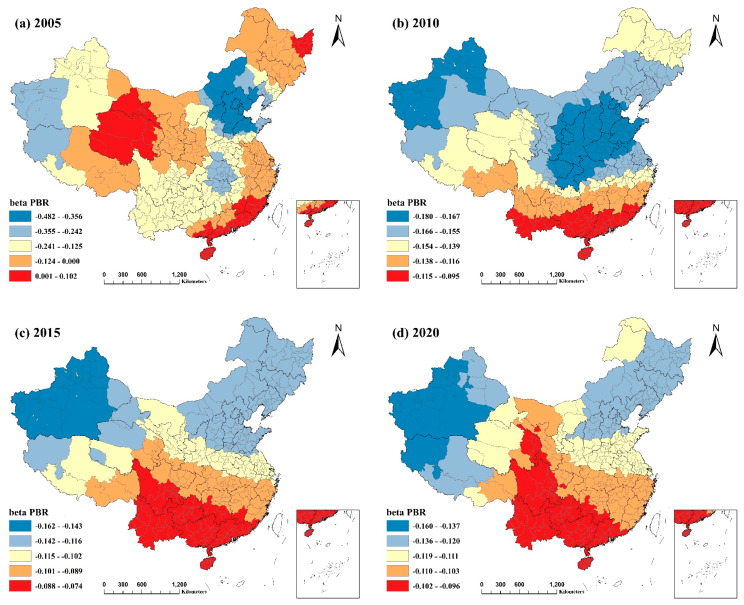
Spatial and temporal distribution of regression coefficients of the general public budget revenue as a proportion of GDP in the MGWR model, 2005−2020: (**a**) spatial and temporal distribution of regression coefficients for the general public budget revenue as a proportion of GDP in 2005; (**b**) spatial and temporal distribution of regression coefficients for the general public budget revenue as a proportion of GDP in 2010; (**c**) spatial and temporal distribution of regression coefficients for the general public budget revenue as a proportion of GDP in 2015; (**d**) spatial and temporal distribution of regression coefficients for the general public budget revenue as a proportion of GDP in 2020.

**Table 1:** Diagnostic results of multicollinearity in explanatory variables.

Explanatory Variables	Units	2005 VIF	2010 VIF	2015 VIF	2020 VIF
GDP per capita (GDPP)	Yuan	2.195	1.755	1.583	1.860
The proportion of secondary industry (SIP)	%	1.580	1.397	1.239	1.348
The number of industrial enterprise units above the scale (NOIE)	individual	2.214	1.915	1.986	1.922
The general public budget revenue as a proportion of GDP (PBR)	%	1.309	1.139	1.101	1.164
Population density (PD)	Person/km^2^	1.532	1.533	1.625	1.554

**Table 2:** China Regional PM2.5 Concentration Global Moran’s I.

Year	Moran’s I	E[I]	SD	z-Value	*p*-Value
2005	0.849	−0.003	0.034	25.394	0.001
2010	0.802	−0.003	0.034	24.008	0.001
2015	0.828	−0.003	0.034	24.869	0.001
2020	0.775	−0.003	0.033	23.571	0.001

**Table 3:** China regional PM2.5 concentration global Moran index.

Explanatory Variables	Coefficients 2005	Coefficients 2010	Coefficients 2015	Coefficients 2020
GDPP	−3.22 × 10^−4 ***^	-7.02 × 10^−5^	−3.95 × 10^−5^	6.28 × 10^−6^
SIP	0.460 ***	0.354 ***	0.341 ***	0.175 ***
NOIE	−1.10 × 10^−4^	−3.66 × 10^−5^	0.002 **	−0.001 ***
PBR	−1.342 ***	−1.424 ***	−0.929 ***	−0.233
PD	0.030 ***	0.026 ***	0.020 ***	0.014 ***

*** Passed the 1% confidence level test, ** passed the 5% confidence level test.

**Table 4:** OLS model fit results.

Year	R^2^	Adjusted R^2^	AIC	F-Statistic	Jarque–Bera Test	BP Test
2005	0.478	0.470	2825.5	64.733 ***	242.300 ***	494.770 ***
2010	0.371	0.362	2931.1	41.681 ***	338.290 ***	225.674 ***
2015	0.337	0.328	2938.9	36.146 ***	33.749 ***	144.421 ***
2020	0.160	0.149	2810.6	13.755 ***	462.666 ***	85.517 ***

*** Passed the 1% confidence level test.

**Table 5:** Regression results for GWR and MGWR models.

Model	Year	R^2^	Adjusted R^2^	AICc	RSS
GWR	2010	0.822	0.780	568.304	64.063
	2010	0.822	0.780	568.304	64.063
	2015	0.861	0.818	537.818	50.168
	2020	0.776	0.724	664.103	82.121
MGWR	2005	0.919	0.898	308.001	29.008
	2010	0.866	0.837	455.921	48.243
	2015	0.884	0.858	408.854	41.908
	2020	0.805	0.771	568.418	71.545

GWR: geographically weighted regression, MGWR: multiscale geo-weighted regression.

**Table 6:** Variable bandwidths for GWR and MGWR models.

Model	Year	Variable Bandwidths				
GWR	2010	54				
	2010	69 54				
	2015					
	2020	70				
MGWR		GDPP	SIP	NOIE	PBR	PD
	2005	359	53	43	53	46
	2010	335	71	43	278	43
	2015	135	86	43	337	43
	2020	329	72	353	353	45

GDPP: GDP per capita, SIP: secondary industry proportion, NOIE: number of industrial enterprise units above the scale, PBR: general public budget revenue as a proportion of GDP, PD: population density.

## Data Availability

The data presented in this study are available on request from the corresponding author.
